# Editors as allies: Our two-year experience at *PLOS Global Public Health*

**DOI:** 10.1371/journal.pgph.0002644

**Published:** 2023-11-27

**Authors:** Julia Robinson, Catherine Kyobutungi, Zena Nyakoojo, Madhukar Pai

**Affiliations:** 1 Public Library of Science, San Francisco, California, United States of America; 2 African Population and Health Research Center, Nairobi, Kenya; 3 McGill School of Population and Global Health, McGill University, Montreal, Quebec, Canada

Two years ago, *PLOS Global Public Health* began publishing articles with a bold vision [1]:

The mission of *PLOS Global Public Health* is to address deeply entrenched inequities in global health and make impactful research visible and accessible to health professionals, policymakers, and local communities. We are committed to amplifying the voices of underrepresented and historically excluded communities and are deliberate and intentional about equity, diversity, and inclusion at all levels–editors, editorial boards, peer reviewers and authors—to broaden the range and diversity of perspectives we learn from and advance the health of all humankind.

We launched this journal in a time of great turmoil: the COVID-19 pandemic was raging with a woefully inequitable distribution of vaccines, Black Lives Matter and Women in Global Health were advocating for urgent change, and the discourse around decolonizing global health shone a brighter light than ever on the way that global health as usual perpetuates systemic inequities. In sum, there was a need to disrupt the way a journal publishes and presents global health research, and we sought to do just that. We created a journal armed with data [2] on how global health journals are not really global, nor seen as safe spaces for Global South, Black, Indigenous, and people of color (BIPOC). We wanted to create a journal that was in and of itself an ally to these communities and to intersecting movements and to the arc of social justice, and we wanted our editors to serve as allies as well.

We set ourselves ambitious goals, beginning with diverse leadership all the way from our Editors-in-Chief (EICs), but also including our Section Editors and, crucially, our Academic Editors, who would assess and improve the research submitted to *PLOS Global Public Health*. We wanted to “amplify the work of BIPOC experts, especially people from the Global South, Indigenous scholars, and individuals working and living within their impacted communities,” ensuring that our journal was a welcome home for work about the Global South, *by* the Global South, as well as to amplify research about inequities wherever they occur. We reaffirmed our commitment to tackling parachute research and removing article processing charges as a barrier to publishing rigorous, peer-reviewed research.

So two years on, how are we doing? Are we truly, as we sought to be, walking the path of allyship? Are we truly diverse and inclusive? Where can we do better?

A journal is, in many ways, a sum of the people that make it happen and, in this case, we are proud of how diverse and distributed we are. Our 43 Section Editors are from 21 countries, with half from the Global South and half from the Global North [3]. Understanding that women are underrepresented in leadership roles in global health, we intentionally recruited a majority (about three-fourths) of women Section Editors, who are actively guiding our strategy and vision and holding the EICs accountable. We have more than 680 Academic Editors from 76 countries at the time of this writing, and 53% of them are from the Global South [4]. We are proud that we were independently ranked as the most geographically diverse editorial boards of all global health journals [5].

This kind of diverse representation did not happen by accident; we have been relentlessly intentional about recruitment, understanding that the power asymmetries in global health will not automatically right itself without intent and hard work. We want to make sure that the research we publish has been assessed by experts: experts in topic, experts in methodology, and experts in the context in which the research has been conducted.

We pledged to avoid elitism and to “amplify the work of BIPOC experts, especially people from the Global South, Indigenous scholars, and individuals working and living within their impacted communities.” In our first two years of publishing articles, we’ve featured work by corresponding authors from 85 countries, with almost half coming from countries in the Global South. We have intentionally solicited articles from Indigenous experts [6, 7], Black scholars [8, 9], and people with lived experience [10–12]. We are working hard to remove barriers to authorship and publishing, whether through our Global Equity Model [13], our more inclusive authorship policies [14], or our constant outreach by our Editorial Board.

Supporting and amplifying the voices of young people has always been very important to us—they are the next generation of global health leaders and are already leading the field on critical issues such as climate justice and decolonizing global health. We’ve invited young people to write some of our most powerful Opinions and Reviews, including Anpotowin Jensen and Victor A. Lopez-Carmen’s piece on Indigenous nations and white settler colonialism [15], Thilagawathi Abi Deivanayagam and Rhiannon Elizabeth Osborne’s review on breaking free from tunnel vision for climate change and health [16], Shashika Bandara and fellow youth leaders’ powerful Opinion on the negative impact of visa and passport inequities on global health and beyond [17], and Daniel Krugman’s insightful article on elite capture of decolonization [18] among many others.

Two years on, we are also taking a hard look at what we haven’t yet accomplished, and all the ways we can continue to push this journal to tackle other barriers to access. We recognized upon our launch, and have been subsequently reminded by our Section Editors, that publishing only in English is a major obstacle to many researchers doing important work around the world [19]. We currently offer authors the option to submit translations of abstracts or other materials as supplementary information, but we recognize the inadequacies of this approach and are working across the PLOS portfolio to come up with expanded options for multilingual offerings.

Relatedly, we recognize that our reach, while global, is not yet global enough: there are certain regions of the world, such as Latin and South America, Francophone Africa, Asia-Pacific, and others; and certain sections, such as Planetary and Environmental Health, Nursing and Midwifery, Racism and Health, and others, that we could do much better in representing. We want to be truly representative of the field, and our body of work—while something we are fiercely proud of—has not yet reached these levels. We want to improve on that.

We also recognize that parachute research continues to be a concern in global health and are working hard to implement our inclusion policy, and broaden the authorship criteria to go beyond the standard ICJME criteria, which, while important and valid, may not entirely recognize the diversity of people who contribute to global public health research.

We want to take this opportunity of our two-year anniversary to mark our early successes and make a renewed commitment to be editors as allies. To inspire further work by us, our editors, and other journal editors, we offer 10 suggestions on how editors in every area can be better allies. We recognize that allyship is not a destination, rather it is a continual process of hard work, reflection, accountability, and responsiveness.

**10 things editors and journals can do to be better allies**:

1. Diversify editorial boards at all levels (Editors-in-Chief, Section Editors, and Academic Editors)
2. Tackle the paywall problem for low- and middle-income countries and ensure open access to all research
3. Address the article processing charge (APC) barrier for low- and middle-income countries
4. Check if we are platforming the same Global North voices and commit to genuine diversity of authors
5. Intentionally seek out and commission articles from Black, Indigenous, and People of color (BIPOC) experts, especially people from the Global South, Indigenous scholars, and individuals/activists with lived experience
6. Explicitly discourage parachute research
7. Support authorship models that are more inclusive, equitable, and self-reflexive
8. Address barriers to access related to language and publication criteria
9. Shift power to regional/country colleagues; support Global South journals, conferences, and initiatives.
10. Publish brave content that challenges status quo in global health!
